# Phenolic Acid Composition of Coffee Cascara in Connection with Antioxidant Capacity: A Geographic Assessment

**DOI:** 10.3390/antiox14050502

**Published:** 2025-04-22

**Authors:** Ningjian Liang, David D. Kitts, Xiwen Wang, Ziying Hu, Maidinai Sabier

**Affiliations:** 1 Nutrition, College of Health, Oregon State University, Corvallis, OR 97331, USA; 2 Food, Nutrition and Health, Faculty of Land and Food Systems, The University of British Columbia, Vancouver, BC V6T 1Z4, Canada; xiwenw@student.ubc.ca (X.W.); msabier@student.ubc.ca (M.S.); 3 Department of Food Science, McGill University, Québec, QC H9X 3V9, Canada; huziying2000@gmail.com

**Keywords:** cascara, polyphenol profile, antioxidant capacity, PCA

## Abstract

Coffee cascara is an underutilized byproduct of coffee processing that has the potential for value-added applications due to its rich phytochemical content and antioxidant properties. The aim of this study was to characterize the phytochemical composition and antioxidant activity of coffee cascara sourced from seven geographic regions, and where possible, a variety of farms in different regions. We compared two different extraction methods: hot water/sonication-assisted extraction and methanol–water extraction to generate phytochemical content. The antioxidant capacity of extracts was assessed through different assays. Correlations between phytochemical compounds and different antioxidant activities were analyzed first using Pearson’s correlations and then substantiated further using principal component analysis (PCA). The dominant phytochemicals identified in the extracted coffee cascara included gallic acid, chlorogenic acid isomers, mangiferin, protocatechuic acid and rutin. Among the water-extracted samples, the Brazil sample exhibited the highest oxygen radical absorbance capacity (ORAC) value, whereas the Zambia sample had the highest 2,2′-azino-bis (3-ethylbenzothiazoline-6-sulfonic acid) (ABTS) value and the Laos sample showed the greatest inhibition of 2′,7′-Dichlorofluorescein diacetate (DCFH-DA) fluorescence. For methanol extracts, the highest ORAC and ABTS values were from the Indonesia sample, and the Laos sample showed the strongest inhibition of DCFH-DA fluorescence. The results show the distinct phytochemical composition and antioxidant activity of coffee cascara according to geographical clustering using PCA. Specifically, gallic acid, *p*-hydroxybenzoic acid and to a lesser extent rutin correlated (*p* < 0.05) with ABTS and DCFH-DA assays. This study revealed significant variation in the chemical composition and antioxidant properties of coffee cascara across different geographic regions; less so with different farms associated with the location. The findings offer evidence for potential upscaling of coffee cascara waste for use in value-added functional food or nutraceutical applications.

## 1. Introduction

Coffee ranks as one of the most widely consumed beverages worldwide, with increasing global demand; hence, this high demand for coffee beverage generates substantial by-product waste such as coffee cherry skin and pulp, termed cascara [1]. These by-products, if sent to landfills, possess potential environmental risks [2,3]. Coffee cascara is rich in macronutrients such as carbohydrates, soluble fibers, minerals and proteins, thus representing a promising candidate for industrial applications in biosorbents, biodiesel, and animal feed materials [4,5,6]. In addition, coffee by-products contain many potential bioactive polyphenols that could be revalorized for use in the circular economy for functional foods [7,8,9,10]. To lower ecological hazards and recover a potential supply chain of bioactive phytochemicals, attempts have been made to process coffee cherry residues, also referred to as cherry cascara, for sustainable, nutritionally rich ingredients that can be used for functional foods [8,11].

The chemical complexity and variability of coffee cascara composition are influenced by factors such as coffee variety, geographic origin, and processing drying methods, thus restricting its use in applications with consumer health benefits that require a standardized composition of specific bioactive components [9]. Following harvesting, extraction of phytochemicals using polar (water) and semi-polar (methanol) solvents is used to recover a mixture of phytochemical compounds with a collective potential for bioactivity applications [12]. Water extraction follows green technology and is a relatively simple and cost-effective method to extract bioactive compounds from a cascara matrix. Alternatively, methanol solvent extraction is the traditional method used to recover phenolics and flavonoids but may require additional processing to remove unwanted chemical residues. This may affect the relative bioactivity of the final standardized extract [12,13,14].

Previous research has examined extracts derived from coffee cascara, spanning different varieties such as Arabica, Tabi, and Robusta, and sourced from diverse geographic origins for antioxidant potential, as evidenced by a capacity to scavenge radicals using chemical methods such as 2,2′-azino-bis (3-ethylbenzothiazoline-6-sulfonic acid) (ABTS) assay, 2,2-diphenyl-1-picrylhydrazyl (DPPH) assay, and ferric reducing antioxidant (FRAP) assay [11,15,16]. Indication of antioxidant capacity of coffee waste has also been shown to translate into biological studies that have shown diminished cellular oxidative stress in response to Tert-butyl hydroperoxide before treatment [17], mitigation of intracellular oxidation triggered by hydrogen peroxide treatment, and reduced expression of key pro-inflammatory markers (e.g., nitric oxide, tumor necrosis factor-α, interleukin 6) in RAW264.7 macrophages and 3T3-L1 preadipocytes [18]. Furthermore, coffee pulp extracts have exhibited a capacity to counteract the release of the pro-inflammatory marker interleukin 8 in gastric epithelial cells [7].

Limited research exists on how cascara phenolic composition varies across different geographic regions, which is critical for optimizing its potential use in functional food and nutraceutical applications. The objective of this study was therefore to characterize the phytochemical composition of coffee cascara extracts collected from different countries and within multiple farms. We compared using both green and solvent-extraction methods to assess the recovery of phenolic acids and related antioxidant capacity using both chemical and biochemical cellular assays. These analyses are required to determine the potential of supporting cascara revalorization for its use as a value-added ingredient for the formulation of novel products in the circular economy.

## 2. Materials and Methods

### 2.1. Samples

Coffee cascara samples, sourced from seven different geographical locations and some farms within these locations, were collected and provided by Olam International (Singapore). Cascara, a by-product of coffee production, was collected and stored at 40 °C before being ground into a powder using a food-grade grinder. Ground powder samples were stored at −80 °C to preserve them until analyzed.

### 2.2. Proximate Composition

Proximate composition of ground cascara samples collected from each country was determined using two replicates for each sample. Specifically, data for moisture, crude protein (cf. 6.25), crude fiber, crude fat, crude ash, total carbohydrate (determined by difference), and gross energy density were analyzed by SGS Inc. (Burnaby, BC, Canada), according to standard AOAC 2006 [19] procedures.

### 2.3. Water Extraction of Coffee Cascara

Powdered coffee cascara samples (10.0 g) were extracted in triplicate using 3 steps. Initially, samples were extracted twice with 100 mL of boiling water (100 °C) for 15 min and then after cooling, the samples were centrifuged at 3220× *g* for 7 min. The supernatant was collected in a 250 mL volumetric flask. A third extraction of the powder was performed in an ultrasonic water bath for 15 min, followed by centrifugation at 3220× *g* for 10 min. The pooled supernatant was filtered through Whatman No. 4 filter paper and freeze-dried to a powder. Powdered samples were dissolved in ultra-pure water (or cell-medium for culture assay) and filtrated again with the Exapure 0.45 mm nylon filter before use.

### 2.4. Methanol Extraction of Coffee Cascara

The methanol extraction of coffee cascara was performed according to our previously published method for extracting phenolic compounds from coffee beans with modifications [20]. Briefly, one gram of grounded cascara sample was extracted with 70% methanol at 20 °C for 6 h in the dark, using a shaker set to 250 rpm. The mixture was centrifuged at 2900× *g* for 5 min, and the supernatant was collected. The pellet was extracted two more times with 70% methanol and all supernatants were pooled and adjusted to 50 mL using 70% methanol. Volumes were reduced under a vacuum and then freeze-dried to a powder before stored at −80 °C for subsequent analyses.

### 2.5. Analysis of Cascara Polyphenol Composition

Coffee cascara extracts were dissolved in ultra-pure water at concentrations of 10 mg/mL and 5 mg/mL, respectively, and filtered using an Exapure 0.45 μm nylon filter. Samples were also analyzed using high-performance liquid chromatography (HPLC) to quantify individual polyphenol content using an Agilent 1100 LC system, equipped with a Phenomenex Kinetex C18 column and a diode array detector. Separation of phenolics was achieved using a gradient elution protocol consisting of 0.1% trifluoroacetic acid (TFA) in water (solvent A) and acetonitrile (ACN) (solvent B). The injection volume, flow rate and column temperature were 5 μL, 1.5 mL/min, and 25 °C, respectively. The following pure standards (>95% purity) used for quantification were as follows: chlorogenic acid (CGA) isomers including 3-caffeoylquinic acid (3-CQA), 4-caffeoylquinic acid (4-CQA), 5-caffeoylquinic acid (5-CQA), 3,4-dicaffeoylquinic acid (3,4-diCQA), 3,5-dicaffeoylquinic acid (3,5-diCQA), and 4,5-dicaffeoylquinic acid (4,5-diCQA), *p*-coumaric acid, ferulic acid, protocatechuic acid, *p*-hydroxybenzoic acid, gallic acid, quercetin, mangiferin, isomangiferin, troxerutin, rutin and (−)-epicatechin (Sigma-Aldrich. St. Louis, MO, USA). The wavelengths used were as follows: 330, 257, 264, and 280 nm (Figure S2). Chemical standards were made at a concentration of 1 mg/mL in methanol and stored in the dark at −80 °C. On the day of analysis, standard stocks were diluted to 100, 50, 25, 12.5, 6.25, 3.13, and 1.56 μM with ultra-pure water and filtered prior to use.

### 2.6. ABTS Assay

ABTS assays were performed to determine the antioxidant capacity of coffee cascara water and methanol extracts, respectively, according to our previous published method [20]. Briefly, ABTS radical cation (ABTS^+^) was prepared by mixing 7 mM ABTS with 2.45 mM potassium persulfate in distilled water and incubating the solution in the dark at room temperature for 12 to 16 h. On the day of testing, the ABTS^+^ working solution was diluted to reach an absorbance of 0.70 ± 0.02 at 734 nm. For the assay, 20 µL of diluted sample extract, or Trolox standard, was added to 180 µL of ABTS working solution in a 96-well clear microplate. After 6 min of reaction, absorbances were measured at 734 nm using a Multiskan Spectrum microplate reader (Thermo Fischer Scientific, Vantaa, Finland). All measurements were conducted in triplicate. The radical scavenging activity of coffee cascara extracts was determined by calculating the ratio between the slopes of the regression equations for the extracts and the Trolox standard, respectively. The results were expressed as μmol Trolox Equivalent (TE)/g of the sample.

### 2.7. ORAC Assay

The oxygen radical absorbance capacity (ORAC) assay of coffee cascara extracts was performed on cascara extracts using our previously published method [21]. On a black 96-well dark plate, 100 μL of diluted sample extract or Trolox, respectively, was mixed with 60 μL of 200 nM fluorescein sodium salt. The plate was incubated at 37 °C in the dark, and 40 μL of 60 mM AAPH was added to each well. Fluorescence intensities were measured every minute for 60 minutes using a plate reader (Tecan Austria GmbH; Männedorf, Switzerland) with excitation and emission wavelengths of 485 nm and 527 nm, respectively. All measurements were performed in triplicate. The area-under-the-curve (AUC) was calculated using Equation (1) [20]. The antioxidant capacity of extracts was determined from the ratio between the regression equations slopes for extracts and Trolox standard, respectively. ORAC values were expressed as μmol Trolox Equivalent (TE)/g of the sample.

Calculation of Area-Under-curve.

(1) $$AUC=\sum_{n=1}^{60}\frac{f_{n}}{f_{1}}=1+\frac{f_{2}}{f_{1}}+\frac{f_{3}}{f_{1}}+\ldots +\frac{f_{59}}{f_{1}}+\frac{f_{60}}{f_{1}}$$

where *f_n_* is the fluorescence intensity at time *n* [20].

### 2.8. Caco-2 Cell Culture

The human colon adenocarcinoma cell line, Caco-2 (HTB-37, American Type Culture Collection, Manassas, VA, USA), was cultured in complete minimum essential medium (MEM) containing Earle’s salts, supplemented with 10% FBS (Invitrogen, Burlington, ON Canada), 100 U/mL of penicillin and 100 µg/mL of streptomycin as described before [20]. Briefly, cells (passage = 26–37) were maintained in 75 cm^2^ plates (Corning Inc., Corning, NY, USA) at 37 °C in a 5% CO_2_ humidified atmosphere, and the media were changed every 2–3 days. Cultured cells were split (1:5) once they reached confluence using 0.05% trypsin-0.5 mM EDTA (Gibco-BRL). For individual experiments, cells were seeded onto 6-well or 96-well plates (Sarstedt AG & Co., Nümbrecht, Germany) at a density of 1 × 10^5^ cells/cm^2^. Cells were cultured for 21 days with a medium change every 2–3 days to allow for spontaneous differentiation.

### 2.9. Cell Viability Assay

For each test, the viability of Caco-2 was established using the 3-(4,5-dimethylthiazol-2-yl)-2,5-diphenyltetrazolium bromide (MTT) assay. After 24 h inhibition, samples were removed and 0.5 mg/mL MTT was added. After incubating cells for 4 h in the dark, to solubilize the formazan crystal, 10% sodium dodecyl sulphate in 0.01% HCl was added; then, overnight, optical density was recorded at 570 nm using the microplate reader (Thermo Fisher Scientific, Vantaa, Finland).

### 2.10. Intracellular Oxidative Assay

The effect of coffee cherry cascara on peroxyl radical-initiated intracellular oxidation was evaluated in differentiated Caco-2 cells using a 2′,7′-Dichlorofluorescein diacetate (DCFH-DA) probe. Differentiated cells were seeded on a 96-well black plate with a clear bottom (Sarstedt AG & Co., Nümbrecht, Germany) at a density of 1 × 10^5^ cells/cm^2^. Final concentrations of cascara extract (0.25 mg/mL) were dissolved in culture media and filtered to sterilize. For each well, 100 μL of cascara extract was incubated with differentiated cells for 24 h. Thereafter, cascara extracts were replaced and incubated with 5 μM of DCFH-DA to a final volume of 100 μL for 30 min under darkness. DCFH-DA was then removed and 1 mM AAPH, with a final volume of 100 μL, was added to initiate intracellular Caco-2 oxidation. Fluorescence readings (Ex: 485 nm, Em: 527 nm) of culture media were recorded using the Tecan Infinite M200 Pro (Tecan Austria GmbH; Männedorf, Switzerland) after 1 h of incubation. A positive control was used to represent DCFH-DA with an inducer (AAPH); the negative control contained DCFH-DA only. Percentage inhibition was calculated based on Equation (2) [21].

Calculation of percentage fluorescence inhibition.

(2) $$\%\;\mathrm{Inhibition}=\left(1-\frac{f_{sample}-f_{nc}}{f_{pc}-f_{nc}}\right)\times 100\;\%$$

where *f_pc_* is the fluorescence of the positive control; *f_nc_* is the fluorescence of the negative control [21].

### 2.11. Statistics

All data are expressed as mean ± standard deviation (SD). HPLC and antioxidant analysis were analyzed using ANOVA and Bonferroni post hoc test (GraphPad Prism software, version 8.2) to describe significant differences among sample means, set at level *p* < 0.05. Cascara phenolic concentrations and antioxidant activity data were collected from different geographical locations and represent both hot water and methanol extracts, respectively. Pearson’s correlation test was used to determine correlation between phenolic acid contents of water and methanol extracts with antioxidant activities. This analysis was followed by using principal component analysis (PCA) and biplot analysis (SIMCA-P+ 14.1 software (Umetrics AB, Umeå, Sweden).

## 3. Results

### 3.1. Proximate Analysis of Cascara Samples

Proximate analysis (% DW) and corresponding energy value (kCal/100 g DW) of cascara samples from seven different geographic locations are presented in Table 1. There was a relatively close similarity for macronutrient component content between locations, albeit Laos cascara had a relatively higher protein content compared to other locations.

### 3.2. Phenolic Profile of Coffee Cascara Water and Methanol Extracts

Primary phenolic compounds comprising both the coffee cascara hot water and methanol extracts, respectively, included chlorogenic acid (and isomers), protocatechuic acid, gallic acid, *p*-coumaric acid, rutin, mangiferin, and *p*-hydroxybenzoic acid. The phenolic acid concentrations recovered from cascara extracts using hot water and methanol extracts are presented in Table 2, respectively, whereas major flavonoids recovered from the same cascara sources are given in Table 3. In both hot water and methanol cascara extract, chlorogenic acid, protocatechuic acid, rutin and mangiferin were recovered in similar concentrations, whereas gallic acid recovery was higher (*p* < 0.05) in the hot water extract. Trace amounts of *p*-hydroxybenzoic acid, *p*-coumaric acid and iso-mangiferin were also detected in the hot water extracts, but not recovered to the same extent in the methanol solvent. The influence of geographic origin on the phytochemical composition of cascara samples from different countries was also evident, with subtle differences in concentration in most cases. No differences were observed for protocatechuic acid from different countries; however, gallic acid and chlorogenic acid isomers were typically greater in concentration in cascara sampled from Brazil (BA), Peru (P) Indonesia (In) and Zambia (Z) (*p* < 0.05). Both Laos (L) and Boliva (BL) cascara samples were exceptionally good sources of Mangiferin, whereas rutin concentrations were consistently higher in BA and Z sources, respectively. Other notable cascara samples derived from BA and In, respectively, had comparable protocatechuic acid, but markedly higher gallic acid concentrations compared to cascara samples collected from T and Z, respectively. Cascara from BL exhibited comparable mangiferin concentrations to L samples. L and Z samples generally contained higher concentrations of minor phenolic constituents, with the exception of rutin.

### 3.3. Antioxidant Activity of Coffee Cascara Water and Methanol Extract

Table 4 summarizes the different measures of coffee cascara antioxidant activity derived from seven different geographical locations. A comparison of the relative antioxidant activity from different assays using both chemical (ORAC and ABTS) and cell-based (DCFHDA assay) antioxidant assays in same samples is also made between hot water and methanol extractions, respectively. Pearson’s correlation test was performed (Supplement Figure S1) to determine significant associations between phenolic compounds and antioxidant capacities (e.g., ABTS, ORAC, DCFHDA). Of particular interest was the significant correlation between gallic acid and *p*-hydroxbenzoic acid contents (both water and methanol extracts) and intracellular antioxidant capacity (*p* < 0.05) in specific sources of cascara. The ORAC assay identified specific geographical differences in antioxidant capacity for countries that were consistently higher in methanol extracts, compared to hot water extracts (*p* < 0.05) This result corresponded to the phenolic profile for L, BA, P and In. On the other hand, less distinction was obtained between countries that had cascara samples in the ABTS free radical scavenging assay. Despite the lower Trolox value calculated for the ABTS results, compared to ORAC, geographical location, such as L, BA, P and In, exhibited a similar trend with relatively higher radical ABTS scavenging activity. In addition to using chemical free radical-scavenging methods, the in vitro antioxidant capacity of coffee cascara extracts was assessed using a cell-based intracellular assay conducted with a human intestinal epithelium model. In this procedure, differentiated Caco-2 cell monolayers treated with a fluorescent dye, i.e., DCFH-DA, showed intracellular oxidation induced by AAPH by emitting a strong fluorescence signal (*p* < 0.05). The stronger the intracellular antioxidant capacity of the coffee cascara extracts, the lower the Caco-2 emitted fluorescence detected. Consistent with both the chemical assays, methanol extracts in most cases had significantly higher intracellular antioxidant capacity compared to extracts derived from hot water (*p* < 0.05). However, the chemical antioxidant capacity of individual cascara samples from some locations agreed only in part with the Caco-2 intracellular antioxidant result obtained for the same sample. For example, among the cascara water-extracted samples, In and T had a relatively greater capacity to inhibit intracellular oxidative stress in Caco-2 cells, whereas cascara from locations such as BA and In that were extracted using methanol showed similar capacities. The differences between using chemical radical scavenging tests versus biological antioxidant assay to assess cascara antioxidant capacity added a third variable for comparison of antioxidant bioactives.

### 3.4. Principle Component Analysis (PCA)

**Figure 1:**
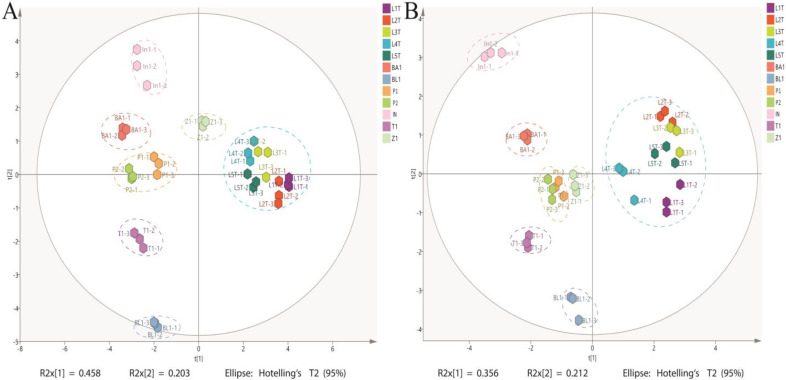
Distribution of samples from different regions, based on total phenolic content and antioxidant activity using coffee cascara extracts, represented by (**A**) score plot of PCA of water extract. (**B**) Score plot of PCA of methanol extract. Geographic location: L = Laos; BA = Brazil; BL = Bolivia; P= Peru; In = Indonesia; T = Tanzania; Z = Zambia. Locations with numbers attached refer to individual farms in the geographic location.

**Figure 2:**
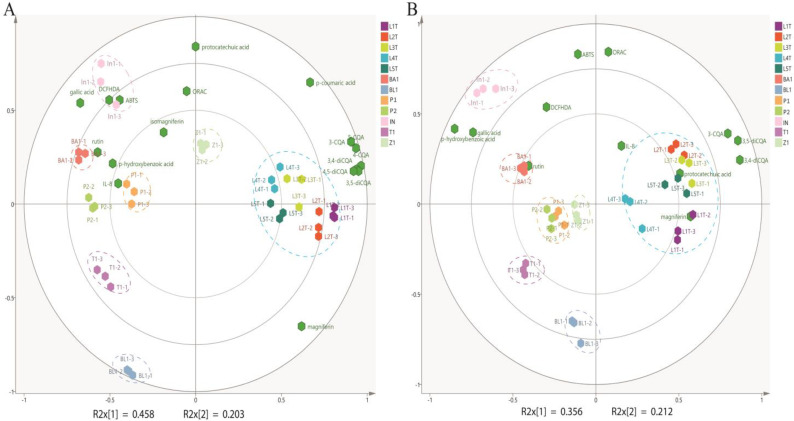
Principal component 2D biplot graph based on the PCA model shown for (**A**) water extract samples and (**B**) methanol extract sample. Geographic location: L = Laos; BA = Brazil; BL = Bolivia; P= Peru; In = Indonesia; T = Tanzania; Z = Zambia. Antioxidant assays, ABTS; ORAC and DCFH-DA. Locations with numbers attached refer to individual farms in the geographic location.

The PCA results shown in Figure 1A,B from different geographical regions can be separated according to phytochemical composition. The explained variance percentages for the vertical and horizontal coordinates of the PCA, calculated by SIMCA+ 14.1 software, exceed 50% (e.g., 6.1% and 56.8%, respectively). Prior analysis using Pearson’s Correlation was conducted to substantiate significant correlations prior to conducting PCA. The distribution patterns of the water and methanol extracts were generally similar, with a few exceptions. Samples from the same region but different farms were clustered closer to each other, compared to samples collected from different geographical regions. For example, cascara samples from Laos were found to cluster together in one quadrant while cascara from geographic locations clustered in different quadrants. Notably, cascara samples derived from In, T, and BL farms were positioned far from each other. Collectively, the data show that the phenolic acid compositions of different cascara samples and related antioxidant activity were strongly correlated with the geographical region and the farm in which they were obtained (Figure 2A,B). For example, phenolic compounds recovered from L cascara, such as chlorogenic acid isomers, 3-CQA, 4-CQA, 5-CQA, 3,4-diCQA, 3,5-diCQA and 4,5-diCQA, were clustered together in a single biplot, markedly different from samples derived from In, BA, P, and T, respectively, that were distributed on the opposite side of the biplot. Additionally, compounds such as isomagniferin, *p*-hydroxybenzoic acid, rutin, and gallic acid were also clustered on left side of the biplot, indicating that these compounds were somewhat common in abundance in farm locations collected from In, BA, P and T, respectively. Cascara from the In location possessed gallic acid, ABTS, and DCFH-DA measurements found in the second quadrant, thus showing a correlation between gallic acid concentration with DCFH-DA, and ABTS antioxidant activities in cascara samples that were particularly rich in gallic acid. In contrast, both T1-1-T1-3 and BL1-1-BL1-3 samples, representing farms for each location located in the fourth quadrant, were without other phenolic acids or antioxidant activity indicators in close proximity. These results provide further detail on the findings that specific geographic locations of cascara contained distinct chemical compositions relative to others, but the relative differences between farms were small.

## 4. Discussion

This study reports for the first time the phytochemical composition and related antioxidant activity of coffee cascara, sourced from seven different geographic regions, with some individual farms included. We also compared water (green) with methanol (chemical) extraction methods to assess composition and antioxidant activity. Major polyphenols identified in coffee cascara included protocatechuic, chlorogenic acid (isomers) and gallic acids, along with magniferin, and lower concentrations of *p*-coumaric, rutin, *p*-hydroxybenzoic acids, and isomagniferin. Other researchers have also reported chlorogenic acid and protocatechuic acid to be primary phenolic acids, along with gallic acid, with the flavonoid rutin present in relatively lower quantities [22]. In general, the phytochemical compositions of water- and methanol-extracted cascara samples were somewhat similar between different geographic sources, with the exception that gallic acid, *p*-coumaric acid and isomagniferin recoveries were consistently higher using the hot water sonication extraction method. Polar phenolic acids (gallic acid, *p*-coumaric acid) and flavonoids (isomagniferins) have greater solubility in a water extraction, which was enhanced further using sonication. Previous studies have reported that methanol–water mixtures are more effective to yield higher recoveries of phenolic acids compared to water extraction [23,24]. Others have, however, reported favorable gallic acid concentrations in plant materials extracted using methanol solvent, but no comparison to hot water with sonication was made [12]. A previous study provided evidence for using hot water as a green alternative to the standard methanol–water protocol to recover bioactive phenolics [25]. This discrepancy between studies may be attributed to differences in solvent ratio (50% methanol–water used by some researchers versus 70% methanol used in this study); and the use of sonication in our study to enhance the release of bound polar phenolics from the cascara matrix. Both gallic acid and chlorogenic acids have been shown to exhibit strong in vitro antioxidant activity in both chemical and cell-based assays [20,26]; hence, optimal recovery of these molecules is critical for assessing true bioactivity.

To assess the relative antioxidant activity of the different cascara sources, both chemical and cell-based techniques were used. In general, ABTS and ORAC antioxidant activities gave values in this study that are aligned with those reported previously [22]. Across cascara samples from different geographic locations, free radical scavenging activity was relevant using both the ABTS and ORAC assays, respectively, indicating that phenolic compounds present in cascara have collective antioxidant activity defined by either donating hydrogen atoms transfer (HAT) or electron transfer (ET), or a combination of both. These mechanisms of free radical sequestering are integrated into a single antioxidant activity when determined in a complex mixture of different phenolic compounds used to assess activity [27,28,29]. The ABTS assay, for instance, does not distinguish precisely between the HAT or ET mechanism, thus resulting in a potential difference in activity trend measured compared to the ORAC assay, which mostly depends on the HAT mechanism. For example, with ORAC, a HAT reaction requires a phenolic compound exhibiting activity to associate with a fluorescent probe to compete with a peroxyl (ROO^•^) radical generated in the reaction, thus yielding a measure based on competitive kinetics. Thus, the area under the curve used in the ORAC reaction that calculates antioxidant capacity includes both antioxidant and prooxidant activity, or a net antioxidant capacity compared to the simpler direct electron transfer used to reduce ABTS^+^•. As a result, antioxidant measurements rarely agree with each other if the mechanism of action used in the chemical assay is different—a situation that is even more relevant when assessing complex food matrices [30,31], such as cascara observed in the present study.

The heterogeneous phenolic composition that comprises different cascara sources reported herein is a plausible factor for the observed variability in antioxidant activity obtained using different antioxidant methods. Specific equimolar mixtures of individual antioxidant phenolic compounds with different structures that possess unique lag phases exemplify characteristic quenching efficiencies attributed to different energies of bond dissociation and steric hindrance in different sample matrices [32] is attributed to different antioxidant activities. From the Pearson correlation coefficient analysis, it can be observed that both gallic acid and *p*-hydroxybenzoic acids, extracted using both water-sonicated and methanol extractions, respectively, were correlated (*p* < 0.05) with ABTS and DCFH-DA analysis (Figure S1). This was best shown in the biplot that showed DCFH-DA and ABTS being close to each other in the same quadrant. Of particular interest was the result from both ABTS^+^• radical scavenging and DCFH-DA intracellular activity that corresponded to a unique mixture of abundant gallic acid and *p*-hydroxybenzoic acid in the In cascara samples, common to both water and methanol extracts, respectively.

We used the differentiated Caco-2 intestinal cell model that responds to peroxyl radical (AAPH)-induced oxidative stress to assess in vitro activity of cascara phenolics to mitigate ROS production. Our findings confirmed those of other studies [11,18]. More recent evidence on an indirect mechanism of phytochemicals to yield an intracellular antioxidant effect is in fact based on the action of metabolites generated in the biological assay system that have a nucleophilic propensity arising from auto-oxidation. Recently, a simple example of this mechanism was reported by the autoxidation product of H_2_O_2_ derived from individual phenolic acids in cell-free systems [26]. With the addition of cells to this reaction, a dramatic decline in extracellular H_2_O_2_ generation occurred, which was associated with a spike in intracellular Nrf-2 cell signaling, a key component for upregulation of cellular antioxidant defense system that enables increased antioxidant capacity [26].

Chemical interactions between different phytochemical components that exist in a plant matrix have been related to antioxidant mechanisms using PCA [33,34]. To investigate the relationship between the unique phytochemical profile of coffee cascara and its antioxidant capacity, PCA was performed for both water- and methanol-extracted samples. The cascara water extract showed evidence of both chemical and bioactive responses, which were positively correlated with gallic acid and intracellular antioxidant activity (DCFH-DA). A similar relationship was found for *p*-hydroxybenzoic acid. These findings point to intracellular signaling being involved in the reported antioxidant capacity, arising from a correlation between gallic acid and *p*-hydroxybenzoic acid mixtures in geographic specific cascara locations, and correlated with intracellular antioxidant capacity [35,36,37]. In other plant sources, specific phenolic acids, such as rosmarinic acid, rutin, and chlorogenic acids, were strongly correlated to the antioxidant parameters [37,38,39,40], and gallic acid has a predisposition to generate hydrogen peroxide as a product of autoxidation [41]. The value of using gallic acid as a chemical marker for identifying antioxidant capacity comes from the connection between gallic acid in the Nrf2 pathway, which relates to the nuclear transcription factor and its regulator Keap1. The Nrf2 is bound to the Nrf2–Keap1 complex under non-stress conditions. Responding to oxidative stress, Nrf2 is released from the complex and translated into the nucleus where its association with an antioxidant response element (ARE) initiates transcription of several antioxidant enzyme genes [42], important to combat oxidative stress.

## 5. Conclusions

The potential of utilizing coffee by-products, such as cascara, holds significant economic and sustainability implications, particularly if these materials are efficiently harnessed to recover valuable phytochemicals for functional food or nutraceutical applications. This study demonstrated that cascara from different geographic locations, corresponding to primary global coffee production regions, shares a common general phytochemical profile while exhibiting distinct variations in specific composition and antioxidant activity. Among the identified compounds, gallic acid, chlorogenic acid isomers, mangiferin, protocatechuic acid, *p*-hydroxybenzoic acid and rutin were present across samples, although only a few in the matrix were correlated with different capacities of antioxidant activity. Notably, gallic acid emerged as a promising biomarker for assessing cascara’s potential for revalorization in a circular economy framework. Furthermore, principal component analysis (PCA) revealed distinct clustering patterns based on geographic origin, highlighting regional influences on phytochemical diversity that included gallic acid. These may include climate-related external factors that influence, for example, soil quality and plant health, impacting the phenolic composition of cascara.

Moving forward, these findings provide a foundation for further research on determining the potential health benefits of cascara in human intervention studies. By integrating cascara into value-added products, a more sustainable and circular approach within the coffee industry would result in reducing agricultural waste while enhancing the economic viability of coffee by-products.

## Figures and Tables

**Table 1:** Proximate analysis composition of the cascara samples collected from different geographical locations ^1^.

Parameter	L	BA	BL	P	In	T	Z
Moisture (%)	20.1 ± 0.4	13.4 ± 0.3	16.2 ± 0.4	17.6 ± 0.7	15.3 ± 0.7	15.1 ± 0.8	13.3 ± 0.7
Protein (%)	10.2 ± 0.2	7.3 ± 0.1	10.7 ± 0.1	7.1 ± 0.1	8.8 ± 0.2	7.2 ± 0.1	9.1 ± 0.1
Fat (%)	3.0 ± 0.1	2.1 ± 0.1	2.2 ± 0.1	2.8 ± 0.1	2.0 ± 0.1	3.0 ± 0.1	1.9 ± 0.1
Carbohydrate (%)	61 ± 5	72 ± 7	65 ± 5	68 ± 3	67 ± 4	67 ± 6	70 ± 4
Crude Fiber (%)	9.0 ± 0.7	10.7 ± 0.7	11.8 ± 0.8	9.3 ± 0.6	12.9 ± 0.6	8.0 ± 0.4	9.9 ± 0.5
Ash (%)	5.8 ± 0.1	6.1 ± 0.1	6.8 ± 0.1	7.4 ± 0.1	6.6 ± 0.1	7.7 ± 0.1	6.3 ± 0.1
Energy (calories/100 g)	315	330	317	326	322	325	327

^1^ Values represent mean ± SD (*n* = 3). Abbreviations of the geographic locations: L = Laos; BA = Brazil; BL = Bolivia; P = Peru; In = Indonesia; T = Tanzania; Z = Zambia.

**Table 2:** Phenolic acids recovered in water and methanol cascara extracts from different geographic locations ^1^.

Code	Protocatechuic Acid		Gallic Acid		*p*-Coumaric Acid ^2^		*p*-Hydroxybenzoic Acid		CGA ^3^	
	Water	Methanol	Water	Methanol	Water	Methanol	Water	Methanol	Water	Methanol
L	2952 ± 47 ^a,x^	2313 ± 84 ^a,x^	1035 ± 31 ^b,x^	56 ± 9 ^c,y^	121 ± 13 ^c^	ND	15 ± 3 ^a,y^	24 ± 4 ^a,x^	8665 ± 62 ^c,x^	7867 ± 242 ^d,x^
BA	3050 ± 38 ^a,x^	3186 ± 49 ^a,x^	7958 ± 111 ^c,x^	884 ± 99 ^a,y^	73 ± 17 ^b^	ND	91 ± 4 ^b,x^	101 ± 13 ^b,x^	3861 ± 1899 ^e,x^	3725 ± 58 ^c,x^
BL	2409 ± 66 ^a,x^	2563 ± 27 ^a,x^	1226 ± 342 ^a,x^	124 ± 14 ^c,y^	17 ± 15 ^a^	ND	125 ± 1 ^b,x^	174 ± 14 ^b,x^	1195 ± 431 ^a,x^	1058 ± 20 ^s,x^
P	2757 ± 18 ^a,x^	2715 ± 25 ^a,x^	4895 ± 120 ^b,x^	101 ± 7 ^c,y^	65 ± 35 ^b^	ND	101 ± 12 ^b,x^	213 ± 8 ^b,c,y^	3597 ± 18 ^b,x^	3529 ± 15 ^b,x^
In	2862 ± 22 ^a,x^	2410 ± 50 ^a,x^	7553 ± 131 ^c,x^	882 ± 128 ^a,y^	121 ± 23 ^c^	ND	231 ± 18 ^c,x^	312 ± 80 ^c,y^	3837 ± 18 ^a,x^	2787 ± 26 ^g,y^
T	2671 ± 14 ^a,x^	2486 ± 30 ^a,x^	849 ± 32 ^d,x^	408 ± 13 ^b,y^	64 ± 3 ^d^	ND	51 ± 13 ^b,x^	49. ± 10 ^a,x^	2047 ± 28 ^a,y^	1394 ± 13 ^s,x^
Z	2983 ± 22 ^a,x^	3160 ± 64 ^a,x^	1173 ± 40 ^b,x^	395 ± 66 ^b,x^	122 ± 19 ^a^	ND	133 ± 11 ^b,x^	233 ± 4 ^b,c,y^	6886 ± 63 ^c,x^	6371 ± 98 ^d,x^

^1^ Values (μg/g sample) represent mean ± SD (n = 5), analysed in triplicate. Different letters (a, b, c, d) denote significant (*p* < 0.05) differences in geographic location using one-way ANOVA followed by LSD tests among different samples, while (x and y) denote significant difference between methanol and water extracts measured from same sample. Geographic location: L = Laos; BA = Brazil; BL = Bolivia; P = Peru; In = Indonesia; T = Tanzania; Z = Zambia. ^2^ *p*-Coumaric acid. N/D = not detected (detection limit < 0.1 μg/g sample in methanol extracts). ^3^ CGA= sum of 3-CQA,4-CQA,5-CQA,3,4-diCQA,4,5-diCQA and 3,5-diCQA chlorogenic isomers.

**Table 3:** Flavonoids recovered in water and methanol cascara extracts from different geographic locations ^1^.

Code ^2^	Mangiferin		Rutin		Isomangiferin	
	Water	Methanol	Water	Methanol	Water	Methanol
BA	2039 ± 25 ^b,x^	2554 ± 27 ^b,y^	250 ± 106 ^c,x^	116 ± 15 ^a,y^	40 ± 7	ND
BL	3107 ± 86 ^c,x^	4294 ± 18 ^a,y^	83 ± 45 ^a,x^	80 ± 2 ^a,x^	21 ± 7	ND
P	1913 ± 22 ^b,x^	2246 ± 10 ^a,x^	164 ± 10 ^b,x^	173 ± 9 ^b,x^	35 ± 7	ND
In	818 ± 12 ^c,x^	2874 ± 70 ^a,y^	147 ± 19 ^b,x^	150 ± 21 ^b,x^	31 ± 4	ND
T	2051 ± 34 ^b,x^	2155 ± 15 ^b,x^	133 ± 51 ^b,x^	187 ± 41 ^b,y^	32 ± 4	ND
Z	2462 ± 44 ^a,b,x^	1555 ± 28 ^c,x^	207 ± 12 ^c,x^	184 ± 21 ^b,x^	30 ± 8	ND

^1^ Values are (μg/g sample) and means ± SD (n = 3). Different letters (a, b, c) denote significant (*p* < 0.05) differences in geographic location using one-way analysis of variance (ANOVA) followed by LSD tests among different samples, while (x and y) denote significant difference between methanol and water extracts measured from the same sample. ^2^ Geographic location: L = Laos; BA = Brazil; BL = Bolivia; P = Peru: In = Indonesia; T = Tanzania; Z = Zambia. ^2^ Isomangiferin. ND = not detected (detection limit < 0.1 μg/g sample in methanol).

**Table 4:** Antioxidant capacity in water and methanol cascara extracts from different geographic locations ^1^.

	ORAC		ABTS		DCFH-DA	
Code ^2^	Water	Methanol ^3^	Water	Methanol ^3^	Water	Methanol ^3^
L	252 ± 13 ^b,x^	418 ± 16 ^c,y^	72 ± 9 ^a,x^	126 ± 7 ^b,y^	20 ± 7 ^a,x^	26 ± 8 ^a,x^
BA	339 ± 5 ^c,x^	364 ± 4 ^b,y^	86 ± 6^a,x^	171 ± 5 ^b,y^	16 ± 10 ^a,y^	36 ± 6 ^b,y^
BL	160 ± 7 ^a,x^	216 ± 12 ^a,y^	61 ± 4 ^a,x^	73 ± 12 ^c,x^	12 ± 11 ^a,x^	16 ± 7 ^a,x^
P	273 ± 19 ^b,x^	403 ± 18 ^c,y^	123 ± 21^b,x^	165 ± 5.0 ^b,x^	14 ± 5 ^a,x^	31 ± 14 ^b,y^
I	252 ± 11 ^b,x^	614 ± 26 ^a,y^	183 ± 9 ^c,x^	293 ± 15 ^a,y^	35 ± 4 ^b,x^	39 ± 9 ^b,x^
T	133 ± 14 ^a,x^	341 ± 24 ^b,y^	105 ± 4 ^b,x^	66 ± 28 ^c,y^	36 ± 7 ^b,x^	19 ± 4 ^a,y^
Z	244 ± 5 ^b,x^	391 ± 33 ^b,y^	208 ± N/A ^c,x^	119 ±14 ^b,y^	26 ±3 ^a,x^	16 ± 6 ^a,y^

^1^ Values are μmol Trolox/g sample for ORAC and ABTS, and percent inhibition for DCFH-DA. Values are means ± SD (n = 3). Different letters (a, b, c) denote significant (*p* < 0.05) differences in geographic location using one-way ANOVA followed by LSD tests, while (x and y) denote significant difference between water and methanol extraction measured from the same sample. ^2^ Geographic location: L = Laos; BA = Brazil; BL = Bolivia; P = Peru; In = Indonesia; T = Tanzania; Z = Zambia. ^3^ one gram of grounded cascara sample was extracted with 70% methanol at 20 °C for 6 h in the dark, using a shaker set to 250 rpm.

## Data Availability

The article’s material includes the original contributions presented in this study. Further enquires can be directed to the corresponding authors upon reasonable request.

## Supplementary Materials

The following supporting information can be downloaded at https://www.mdpi.com/article/10.3390/antiox14050502/s1. Figure S1: Pearson’s correlation coefficients between phenolic acids present in cascara water (A) and methanol (B) extracts with antioxidant activities; Figure S2: Chromatogram showing phenolic acid compound standards used in the study.

## Abbreviations

The following abbreviations are used in this manuscript:

ABTS	2,2′-azino-bis (3-ethylbenzothiazoline-6-sulfonic acid)
ACN	Acetonitrile
BA	Brazil
DCFH-DA	2′,7′-Dichlorofluorescein diacetate
ET	Electron transfer
PCA	Principal component analysis
HPLC	High-performance liquid chromatography
Keap1	Kelch ECH associating protein 1
Nrf2	Nuclear factor-erthyriod 2 -related factor 2
ORAC	Oxygen radical absorbance capacity
HAT	Hydrogen atom transfer
TFA	Trifluoroacetic acid
P	Peru
In	Indonesia
Z	Zambia
L	Laos
BL	Boliva
MTT	3-(4,5-dimethylthiazol-2-yl)-2,5-diphenyltetrazolium bromide
CGA	Chlorogenic acid
3-CQA	3-caffeoylquinic acid
4-CQA	4-caffeoylquinic acid
5-CQA	5-caffeoylquinic acid
3,4-diCQA	3,4-dicaffeoylquinic acid
3,5-diCQA	3,5-dicaffeoylquinic acid
4,5-diCQA	4,5-dicaffeoylquinic acid
