# Supplementary material for: Phenolic Acid Composition of Coffee Cascara in Connection with Antioxidant Capacity: A Geographic Assessment
Source: Antioxidants (Basel). 2025 Apr 22;14(5):502. doi: 10.3390/antiox14050502 (PMC12108463; doi:10.3390/antiox14050502)
Supplement: Supplementary file 1 [file antioxidants-14-00502-s001.zip › antioxidants-3561214-supplementary.pdf]

Supplementary Materials

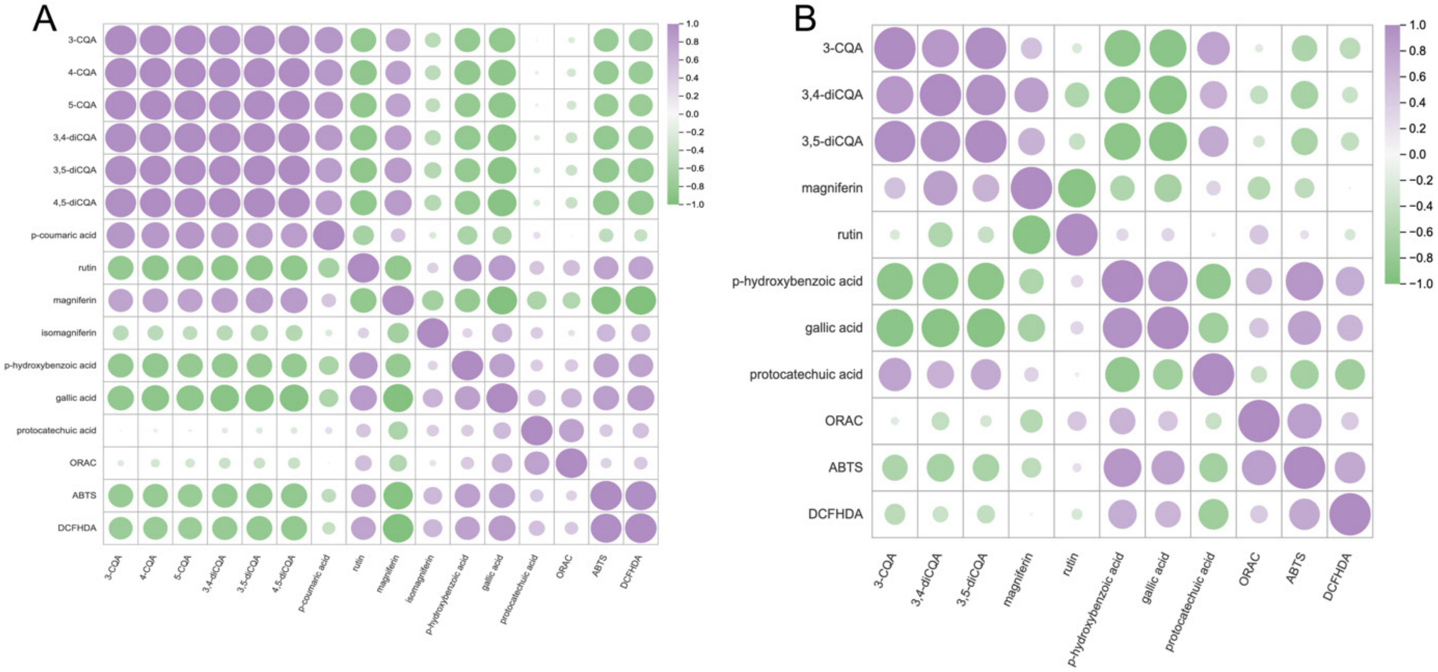

Figure S1: Pearson's correlation coefficients between phenolic acids present in Cascara water (A) and methanol (B) extracts with antioxidant activities.

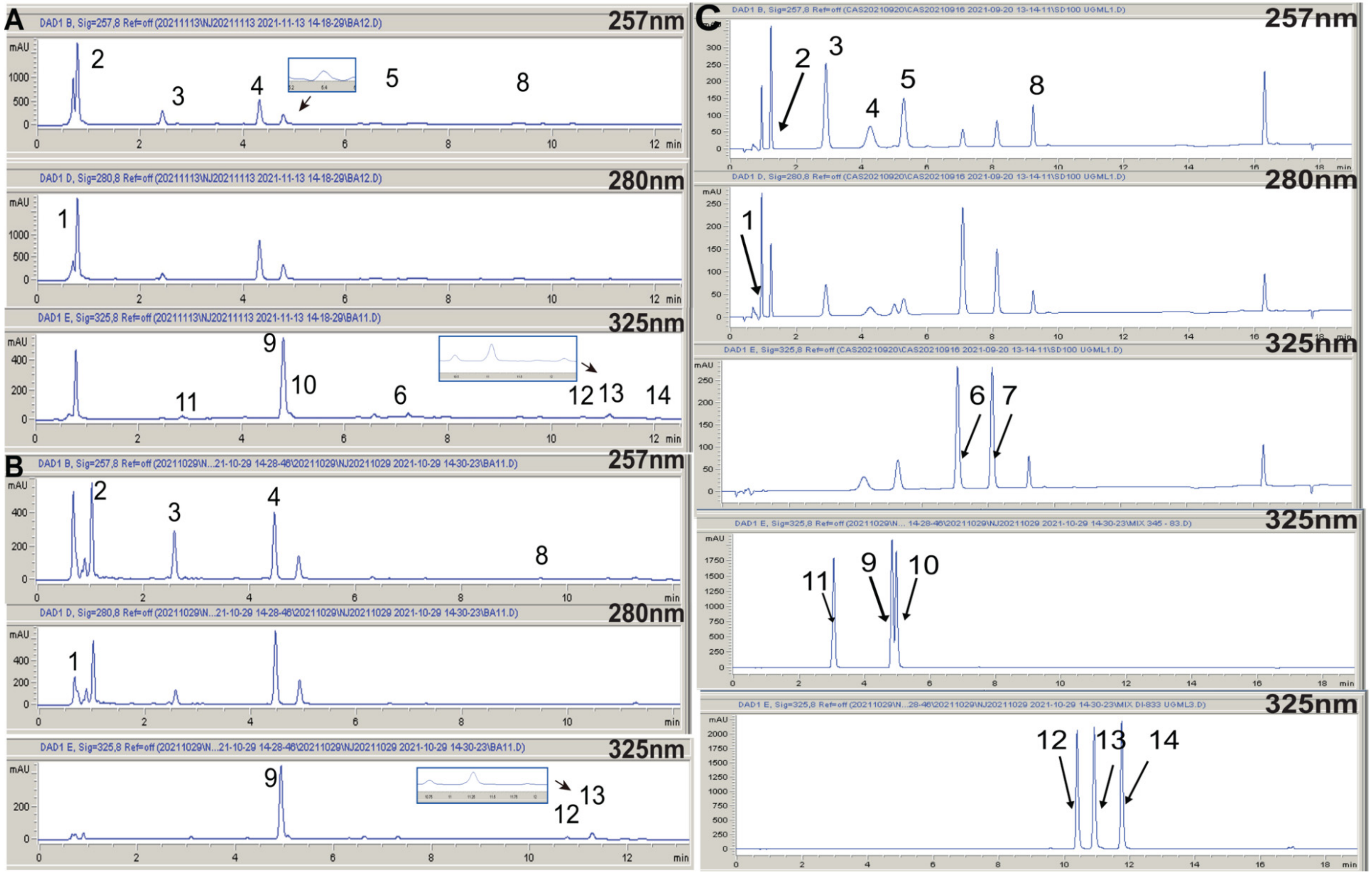

Figure S2. Chromatogram showing phenolic acid compound standards used in the study. Chromatograms showing cascara samples BA water (**A**) and methanol (**B**). Individual phenolic acid composition: 1 = Gallic acid, 2 = Protocatechuic acid, 3 = *p*-hydroxybenzoic acid, 4 = Mangiferin, 5 = Isomangiferin, 6 = Coumaric acid, 7 = ferulic acid, 8 = Rutin, 9 = 3 -CQA, 10 = 4, CQA, 11 = 5-CQA, 12 = 3,4-diCQA, 13 = 3,5-diCQA, 14 = 4,5-diCQA, Inserts represent magnified specific peak response and retention time. (**C**) = standards used to identify phenolic acids in panels A and B.
